# Two-year outcome of half-time photodynamic therapy for chronic central serous chorioretinopathy with and without choroidal neovascularization

**DOI:** 10.1371/journal.pone.0284979

**Published:** 2023-05-02

**Authors:** Aya Kamimura, Akiko Miki, Maya Kishi, Mina Okuda, Mayuka Hayashida-Hirano, Mari Sakamoto, Wataru Matsumiya, Hisanori Imai, Sentaro Kusuhara, Makoto Nakamura

**Affiliations:** 1 Division of Ophthalmology, Department of Surgery, Kobe University Graduate School of Medicine, Kobe, Hyogo, Japan; 2 Department of Ophthalmology, Kobe City Eye Hospital, Kobe, Hyogo, Japan; Yokohama City University, JAPAN

## Abstract

**Purpose:**

To compare the two-year outcome of half-time photodynamic therapy (htPDT) in chronic central serous chorioretinopathy (cCSC) with and without choroidal neovascularization (CNV).

**Methods:**

In this retrospective study, we included 88 eyes of 88 patients with cCSC who underwent htPDT and were followed up for more than 24 months. Patients were divided into two groups with (21 eyes) or without (67 eyes) CNV before htPDT treatment. The best-corrected visual acuity (BCVA), central retinal thickness (CRT), subfoveal choroidal thickness (SCT), and the presence of subretinal fluid (SRF) were evaluated at baseline and at 1, 3, 6, 12, and 24 months after PDT.

**Results:**

A significant intergroup difference was noted in terms of age (*P* = 0.038). Significant improvements in the BCVA and SCT were found at all time points in eyes without CNV but only at 24 months in eyes with CNV. CRT was significantly reduced in both groups at all time points. No significant intergroup differences were noted in terms of BCVA, SCT and CRT at all time points. There were significant differences in the rate of recurrent and persistent SRF between groups (22.4% (without CNV) vs. 52.4% (with CNV), P = 0.013, and 26.9% (without CNV) vs. 57.1% (with CNV), P = 0.017, respectively). The presence of CNV was significantly associated with the recurrence and persistence of SRF after initial PDT (P = 0.007 and 0.028, respectively). Logistic regression analyses showed that the baseline BCVA, and not the presence of CNV, was significantly associated with BCVA at 24 months after initial PDT (P < 0.01).

**Conclusions:**

A htPDT for cCSC was less effective in eyes with CNV than in those without CNV regarding the recurrence and persistence of SRF. Additional treatment might be required in eyes with CNV during 24-month follow-up periods.

## Introduction

Central serous chorioretinopathy (CSC) causes retinal detachment, typically involving the macula, caused by the accumulation of subretinal fluid (SRF) accumulation [1]. Although CSC typically resolves spontaneously within 3–6 months [2] treatment administration should be considered to ensure the preservation of the quality of vision when SRF accumulation persists beyond this period.

Previous studies have demonstrated that half-time photodynamic therapy (htPDT; PDT with standard-dose verteporfin [6 mg/mm2] and halved laser irradiation time [42 s]) is effective, as well as half-dose PDT (hdPDT; PDT with half-dose verteporfin [3 mg/mm^2^] and standard laser irradiation time [83 s]) in treating chronic CSC (cCSC) patients [3, 4]. However, some patients experience persistent or recurrent SRF accumulation after the initial application of htPDT or hdPDT [5, 6]. Several factors, such as the lower baseline best-corrected visual acuity (BCVA), older age, male sex, bilateral disease, and genetic factors are reported to be associated with the recurrence or persistence of SRF after PDT treatment in CSC eyes [7–10].

Previous studies have reported choroidal neovascularization (CNV) in CSC eyes [11], with an incidence ranging from 15.6% to 34.5% [12, 13]. Older age, cCSC, and foveal leakage points on fluorescein angiography were reported as contributive factors in the later development of CNV in CSC eyes [14]. Several studies have reported PDT treatment for CNV in CSC eyes [15–19]. However, there is no report on the long-term outcome of htPDT for CSC with and without CNV, and also none that compares the outcome of htPDT between CSC eyes with or without CNV.

In the present study, we investigated and compared the two-year treatment outcome of htPDT for cCSC with and without CNV.

## Materials and methods

The protocol of this study adhered to the tenets of the Declaration of Helsinki and was approved by the institutional review board of Kobe University Hospital. Also, informed consent was obtained via the opt-out method on the center’s website.

### Participants

We retrospectively reviewed 88 eyes of 88 consecutive patients with cCSC after htPDT who were treated and observed between April 2012 and December 2020 at Kobe University Hospital. The definition of cCSC was based on the PLACE trial [20]. (1) presence of subretinal fluid involving the fovea on optical coherence tomography (OCT) images, (2) active leakage and window defects on fluorescein angiography (FA), and (3) hyperfluorescence on indocyanine green angiography (ICGA). In addition, cCSC was defined as the presence of SRF for at least 6 months after CSC diagnosis, or the continued presence of symptoms that could be attributed to CSC for at least 6 months. Our inclusion criteria was to observe at least a two years of follow-up after htPDT. The exclusion criteria were (1) the presence of any other ocular diseases that could affect visual acuity, including tilted disk syndrome, a dome-shaped macula, and glaucoma, and (2) a previous history of PDT or anti-vascular endothelial growth factor. In the case of bilateral CSC, the right eye was adapted for analysis.

### Treatment

Patients received htPDT according to a protocol identical to that used in previous reports [1, 21]. All patients received an infusion of verteporfin (Visudyne; Novartis, Basel, Switzerland) at 6 mg/m^2^ body surface area over 10 min; laser treatment was administered 15 min after the initiation of infusion. The standard light intensity was 600 mW/cm^2^, and the irradiation time was shortened to 42 s (half-time PDT). The spot size covered the areas with active leaking spots on FA images and the areas with CVH on ICGA images. The spot size covered only the areas defined by FA images in case without CVH. Additional PDT was considered for eyes with persistent or recurrent SRF at the physician’s discretion.

### Baseline and follow-up examination

Patients were assessed at baseline and at 1, 3, 6, 12, and 24 months after PDT. Clinical data regarding age, sex, history of steroid, and the duration of symptoms were collected. In all patients, we performed best-corrected visual acuity (BCVA) measurements, slit-lamp examination, dilated fundus examination, and OCT at each visit.

The Spectralis OCT system (Heidelberg Spectralis OCT; Heidelberg Engineering GmbH, Heidelberg, Germany) was used for obtaining macular scans and for FA and ICGA. Macular 6-line radial and 25-line raster scan with enhanced depth imaging was performed in all patients to evaluate neurosensory retinal detachment, choroidal abnormalities, and PED at baseline. Macular 6-line radial scan with enhanced depth imaging was performed in all patients to evaluate neurosensory retinal detachment and choroidal abnormalities during follow-up. The subfoveal choroidal thickness (SCT), central retinal thickness (CRT), and subretinal fluid (SRF) were evaluated at each visit. The SCT and CRT were manually measured according to previous reports [8]. CRT was manually measured from the outer surface of the neurosensory retina to the inner surface of retinal pigment epithelium (RPE). Additionally, SCT was manually measured from the outer surface of RPE to the inner surface of the sclera.

The recurrence and persistence of SRF were defined according to a previous report [8]. Briefly, ‘recurrence’ was defined as the appearance of SRF after complete absorption following PDT treatment. ‘Prolongation’ was defined as SRF lasting more than three months after the initial PDT treatment.

The presence of CNV was assessed using FA and ICGA. The available cases were evaluated using OCT angiography 3X3 mm and 6X6 mm macula scans (Avanti^®^ RTVue 100 XR OCT system (Optovue, Inc., Fremont, CA, USA) or Cirrus HD-OCT (Carl Zeiss, Dublin, CA)).

SRF recurrence and persistence and the presence of CNV was diagnosed independently by 2 masked retina specialists (A.K., and A.M.). Discrepancies were adjudicated by a third masked retinal specialist (M.K).

### Statistical analysis

Statistical comparisons were performed between the presence of CNV at baseline using chi-squared test or Fisher’s exact test for categorical variables and the Mann–Whitney test for quantitative variables. The decimal visual acuity was converted into log MAR units for statistical analyses. A multiple logistic regression analysis was performed to investigate the factors associated with the recurrence and persistence of SRF after the initial PDT treatment using variables which were reportedly associated with SRF recurrence and persistence. A multivariate analysis was performed to detect the factors associated with BCVA two years after PDT. The changes in logMAR BCVA and OCT findings (SCT, CRT) before and after htPDT during the two-year follow-up period in each group (with/without CNV) were analyzed using the Wilcoxon signed-rank test.

Statistical analyses were performed using SPSS (version 24.0, IBM Corp., Armonk, NY, USA) and MedCalc (version 16.8.4, MedCalc Software, Mariakerte, Belgium). A *P*-value of <0.05 was considered statistically significant.

## Results

Eighty-eight eyes of 88 patients were included in this study. The baseline characteristics of both groups are summarized in Table 1.

**Table 1:** Patients’ demographic and clinical characteristics.

	Total	With CNV	Without CNV	*P*
Number of eyes (n)	88	21	67	-
Sex, male/female	70/18	16/5	54/13	0.758
Age (years)	55.7 ± 13.4	60.5 ± 12.2	54.2 ± 13.4	0.038
Bilateral / Unilateral	12/76	3/18	9/58	1.000
Steroid use, +/−	7/81	1/20	6/61	1.000
Duration (months)	11.9 ± 17.2	11.0 ± 14.1	12.2 ± 18.1	0.612
CVH on ICGA, +/−	79/7	20/1	59/6	1.000
Baseline BCVA (logMAR)	0.145 ± 0.276	0.141 ± 0.157	0.146 ± 0.304	0.281
Spot size PDT (μm)	4174.1 ± 1189.7	4178.6 ± 1210.8	4172.7 ± 1192.3	0.789
Number of PDT (n)	1.5 ± 0.8	1.8 ± 1.1	1.3 ± 0.6	0.059
SCT at baseline (μm)	390.9 ± 113.7	375.8 ± 74.5	395.2 ± 122.8	0.807
CRT at baseline (μm)	313.0 ± 129.1	295.8 ± 109.3	317.9 ± 134.6	0.654

*CNV* choroidal neovascularization, *CVH* choroidal vascular hyperpermeability, *ICGA* indocyanine green angiography, *BCVA* best-corrected visual acuity, *logMAR* logarithm of the minimum angle of resolution, *PDT* photodynamic therapy, *SCT* subfoveal choroidal thickness, *CRT* central retinal thickness. Continuous variables are presented as the mean ± standard deviation.

There were no significant differences in the sex ratio, bilateral, steroid use, duration of symptoms, spot size, the presence of choroidal vascular hyperpermeability, baseline BCVA, CRT, and SCT between eyes with (21 eyes) and without (67 eyes) CNV, except for age (P = 0.038).

### BCVA

**Fig 1:**
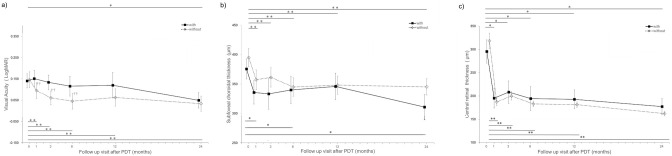
Changes in the Best-corrected Visual Acuity (a), Central Retinal Thickness (b), and Subfoveal Choroidal Thickness (c) during the 24-month Follow-up Period in Eyes With or Without Choroidal neovascularization.

The BCVA in eyes without CNV was significantly improved at 3, 6, 12, and 24 months (all P < 0.05) compared with baseline after PDT, whereas BCVA in eyes with CNV was significantly improved only at 24 months (P < 0.05; Fig 1a). No significant intergroup differences were noted at all time points.

### CRT and SCT

The CRT in both groups significantly decreased at 1, 3, 6, 12, and 24 months compared with the baseline after PDT (*P* < 0.05 at all time points in eyes with and without CNV; Fig 1b). No significant differences were found in CRT at all time points between the two groups.

The SCT in eyes without CNV was significantly decreased at all time points (P < 0.05 at all time points), whereas the SCT in eyes with CNV was significantly decreased only at 24 months (P < 0.05; Fig 1c). There were no significant differences in SCT at all time points between the two groups.

The black squares indicate eyes with CNV and the white diamond indicates eyes without CNV.

1. The BCVA in eyes without CNV improved significantly at 3,6,12, and 24 months compared with the baseline, whereas the BCVA in eyes with CNV improved significantly only at 24 months (Wilcoxon signed-rank test with Bonferroni correction, * eyes with CNV, ** eyes without CNV; both P<0.05). There were no significant differences between the two groups at all time points (Mann–Whitney U test with Bonferroni correction).
2. The SCT in eyes without CNV significantly decreased at all time points, whereas this same parameter in eyes with CNV significantly decreased only at 24 months (Wilcoxon signed-rank test with Bonferroni correction, * eyes with CNV, ** eyes without CNV; both P<0.05). There were no significant differences in SCT at all time points between the two groups (Mann–Whitney U test with Bonferroni correction).
3. The CRT in both groups significantly decreased at all time points (Wilcoxon signed-rank test with Bonferroni correction, * eyes with CNV, ** eyes without CNV; both P<0.05). No significant differences in CRT were found at all time points between the two groups (Mann–Whitney U test with Bonferroni correction).

### Complete resolution of SRF

Almost all patients (61 of 67 eyes, 91.0%) experienced complete resolution of SRF at 24 months after PDT in eyes without CNV. On the other hand, 76.2% (16 of 21 eyes) of patients experienced complete resolution at 24 months in eyes with CNV. However, we did not identify any significant difference in the proportion of patients with complete resolution of SRF at 24 months between the two groups (p = 0.615, chi-squared test with Bonferroni correction).

### Recurrence and persistence of SRF

Eleven out of 21 patients (52.4%) having eyes with CNV and 15 out of 67 patients (22.4%) having eyes without CNV experienced a recurrence of SRF after initial PDT, and the difference in this proportion between the two groups was statistically significant (P = 0.013; Table 2). Twelve out of 18 patients (57.1%) having eyes with CNV and 18 out of 70 patients (26.9%) having eyes without CNV experienced SRF persistence after the initial PDT, and the difference in this proportion between the two groups was statistically significant (P = 0.017; Table 2).

**Table 2:** The rate of recurrence and prolongation in eyes with and without CNV.

	With CNV (n = 21)	Without CNV (n = 67)	*P* *
Recurrence, n (%)	11 (52.4)	15 (22.4)	0.013
Persistence, n (%)	12 (57.1)	18 (26.9)	0.017

*CNV* choroidal neovascularization.

* chi-squared test.

Next, we evaluated the factors associated with the recurrence and persistence of SRF using logistic regression analysis. The presence of CNV was significantly associated with the recurrence and persistence of SRF after the initial PDT during the two-year follow-up period (*P* = 0.006 and 0.024, respectively; Tables 3 and 4). Moreover, bilateral disease was significantly associated with the recurrence (*P* = 0.017).

**Table 3:** Baseline clinical factors associated with recurrence after initial PDT.

Variable	Standard coefficient (β)	*P*
Age (yrs)	−0.056	0.612
Sex (+/−)	−0.048	0.654
Baseline BCVA (logMAR)	0.158	0.125
Bilateral/unilateral	0.252	0.017
CNV (+/−)	0.288	0.006

*PDT* photodynamic therapy, *BCVA* best-corrected visual acuity, *logMAR* logarithm of the minimum angle of resolution, *CNV* choroidal neovascularization.

**Table 4:** Baseline clinical factors associated with persistence after initial PDT.

Variable	Standard coefficient (β)	*P*
Age (yrs)	0.112	0.334
Sex (+/−)	−0.067	0.543
Baseline BCVA (logMAR)	0.013	0.903
Bilateral/unilateral	0.031	0.774
CNV (+/−)	0.246	0.024

*PDT* photodynamic therapy, *BCVA* best-corrected visual acuity, *logMAR* logarithm of the minimum angle of resolution, *CNV* choroidal neovascularization.

We also evaluated the factors associated with BCVA at 24 months using multivariate analysis. The baseline BCVA, not the presence of CNV, was significantly associated with BCVA at 24 months (P < 0.01; Table 5).

**Table 5:** Clinical factors associated with BCVA 24 months after PDT.

Variable	Standard coefficient (β)	*P*
CNV (+/−)	0.032	0.620
Baseline BCVA (logMAR)	0.810	<0.01

*BCVA*, best-corrected visual acuity, *PDT* photodynamic therapy, *CNV* choroidal neovascularization, *logMAR* logarithm of the minimum angle of resolution.

### Complications

There were no patients who experienced a permanent decline of visual acuity, choroidal ischemia, and RPE atrophy after PDT treatment during the follow-up period. However, one patient with CNV exhibited macular hemorrhage one month after the initial treatment. After three consecutive intravitreal injections of ranibizumab (Lucentis; Novartis, Basel, Switzerland), macular hemorrhage was resolved. The remaining patients were treated with half-time PDT at recurrence. No eyes without CNV group developed CNV during the follow-up period.

## Discussion

In this study, htPDT was less effective in cCSC eyes with CNV than in those without CNV regarding the recurrence of SRF. To the best of our knowledge, this is the first report on the two-year treatment outcome of htPDT in cCSC eyes with and without CNV, and it is also the first study to compare the treatment outcomes of htPDT for CNV in cCSC eyes with or without CNV.

The rate of CNV (23.8%; 21 out of 88 eyes) in the present study was comparable with those reported by previous reports (15.6% and 34.5%) [14, 15]. A previous study [12] compared CSC eyes with and without CNV and demonstrated that there were significant differences in BCVA but not in age. In our study, there was a significant difference only in age but not BCVA between eyes with and without CNV. This discrepancy is due to the exclusion of acute CSC in our study. Though acute CSC is typically restored, VA in eyes with cCSC is often severely reduced [1, 2].

Regarding the treatment outcome of htPDT in CSC eyes, Tsai MJ and Hsieh YT [3] reported 16 acute and cCSC eyes treated with FA-guided htPDT. After a mean follow-up period of 12.1 months, the BCVA was significantly improved and all eyes showed complete resolution of SRF. Another study by Liu H et al. [4] compared the efficacy of hdPDT to that of htPDT in acute and cCSC eyes. The BCVA of both groups showed significant improvements at 3, 6, and 12 months after PDT. All 26 eyes which received htPDT showed complete resolution of SRF after the initial PDT whereas two of 26 eyes (7.7%) experienced a recurrence of SRF during the mean follow-up period of 14.8 months. Consistent with the findings of previous studies [4, 5], the BCVA in eyes without CNV improved significantly at 3,6,12, and 24months during the 24-month follow-up in the present study. On the other hand, 15 out of 67 patients (22.4%) having eyes without CNV experienced SRF recurrence, while 18 out of 67 patients (26.9%) having eyes without CNV experienced SRF persistence after the initial PDT. The rates of both recurrence and persistence are higher in our study than in previous reports [3, 4]. The reasons for this discrepancy may include the number of study participants, the follow-up period, and the laser intensity of the PDT instrument.

Chan W.M., et al. [18] reported the outcome of PDT for CNV in CSC eyes, and that the mean BCVA improved significantly at a mean follow-up duration of 12.6 ± 4.9 months and the mean number of PDT sessions was 1.9. A previous report by Hu, Y.C., et al. [22] investigated the three-year treatment outcome of hdPDT in CSC eyes with CNV detected by OCT angiography and reported that the BCVA improved significantly while CRT and SCT decreased significantly with mean 1.5 ± 0.75 PDT sessions. Consistent with the findings of previous studies, the BCVA improved significantly at 24 months after initial PDT while CRT and SCT decreased significantly in eyes with CNV in our study. The mean number of PDT sessions in our study was 1.8, a finding that was consistent with those of the previous study by Chan, W.M., et al. [18], whereas it was higher than the value reported by Hu, Y.C., et al [22]. This difference can be explained by the fact that 12 out of 32 eyes received intravitreal injections of anti-vascular endothelial growth factor at the recurrence or persistent of SRF in the previous study [22].

In the present study, the presence of CNV is the independent factor associated with recurrent or persistent SRF. Among eyes with CNV, the percentage of eyes that underwent more than one session of PDT was about 40% in previous studies [18, 19]; however, it was 48% in our study. Based on these results, the treatment response with reduced setting PDT such as htPDT and hdPDT is not sufficient for the complete resolution of fluid in CSC eyes with CNV. Therefore, another initial therapy should be considered. A previous study [23] demonstrated that approximately 50% of CSC eyes with CNV required additional treatment after intravitreal anti-VEGF injection combined with half-fluence PDT. On the other hand, intravitreal anti-VEGF injection combined with standard PDT required a few additional treatments [24]. In our study, one patient with CNV exhibited macular hemorrhage 1 month after the initial treatment. Occlusion of the choriocapillaris after PDT is suggested to cause the production of VEGF, resulting in the activation of CNV. In this point of view, combination therapy will be desired for eyes with CNV.

Bilateral disease was associated with the recurrence of SRF in our study, which was consistent with a previous study by Lai et al. [9]. Haga et al. [10] reported that low baseline BCVA values and advanced age were associated with recurrent or persistent SRF among 79 cCSC-affected eyes treated with hdPDT. Similarly, Rijssen et al. [7] found that low baseline BCVA and older age, as well as the male sex, were associated with recurrent or persistent SRF among 46 cCSC-affected eyes treated with hdPDT. In the present study, the presence of CNV, but not low BCVA, age or sex, is significantly associated with the recurrence and persistence of SRF. Since patients with CNV were significantly older than those without CNV in our study, previous studies [10] might include eyes with CNV. Li et al. [25] reported that larger flat irregular pigment epithelium detachment was a predictor of anatomic outcome at 3 months after half-dose PDT. Regarding flat irregular PED, Bousquet et al. [26] found CNV in one-third of the flat irregular PEDs in cCSC. It is possible that eyes with CNV were included in the study by Li et al [25].

There were significant differences in BCVA between eyes with and without CNV at 1, 3, and 6 months after the initial treatment. This is because the rate of persistent SRF was higher in eyes with CNV than in eyes without CNV. At 24 months, there were no significant differences in BCVA, CRT, and SCT between eyes with and without CNV, with additional PDT treatment. Moreover, we found that the baseline BCVA, not the presence of CNV, was the independent factor associated with the BCVA at 24 months. Haga et al. [10] demonstrated that the BCVA three years after PDT was positively associated with the baseline BCVA. Though the follow-up period of our study (two years) was different from that of their study (three years), the results of both studies were comparable. In eyes with better BCVA at baseline, functional improvement can be achieved even when CNV was present with appropriate additional treatment 2 years after initial htPDT treatment.

The limitations of this study include the relatively small sample size, the retrospective design, and the fact that additional treatment was performed at the physician’s discretion. Additionally, because OCT angiography was only used in the available cases, the presence of CNV may have been underestimated. Further studies are required to elucidate the treatment effect of htPDT for CSC eyes with and without CNV.

In conclusion, htPDT for cCSC was less effective in eyes with CNV than in those without CNV regarding the recurrence and persistence of SRF. The baseline BCVA was associated with the BCVA at 24 months after initial treatment. Additional treatment might be required in eyes with CNV during the 24-month follow-up periods.
